# Clinical and Phenomenological Characteristics of Patients with Task-Specific Lingual Dystonia: Possible Association with Occupation

**DOI:** 10.3389/fneur.2017.00649

**Published:** 2017-12-11

**Authors:** Kazuya Yoshida

**Affiliations:** ^1^Department of Oral and Maxillofacial Surgery, National Hospital Organization, Kyoto Medical Center, Kyoto, Japan

**Keywords:** lingual dystonia, oromandibular dystonia, tongue, task-specificity, occupation, occupational dystonia

## Abstract

**Background:**

Lingual dystonia is a subtype of oromandibular dystonia, which is a movement disorder characterized by involuntary sustained or intermittent contraction of the masticatory and/or tongue muscles. Lingual dystonia interferes with important daily activities, such as speaking, chewing, and swallowing, resulting in vocational and social disability.

**Objective:**

The aim of this study was to investigate a possible relationship between occupation and the development of lingual dystonia.

**Methods:**

Phenomenological and clinical characteristics of 95 patients [53 females (55.8%) and 42 males (44.2%), mean age 48.0 years] with task-specific, speech-induced lingual dystonia were analyzed. Structured interviews were carried out to obtain information regarding primary occupation, including overtime work and stress during work. The factors that might have influenced the development of lingual dystonia were estimated using multivariate logistic regression analysis of the 95 patients with lingual dystonia and 95 controls [68 females (71.6%) and 27 males (28.4%), mean age 47.2 years] with temporomandibular disorders.

**Results:**

Overall, 84.2% of the patients had regular occupations; 73.8% of the patients with regular occupations reported working overtime more than twice a week, and 63.8% of them experienced stress at the workplace. Furthermore, 82.1% of the patients had engaged in occupations that required them to talk to customers or other people under stressful situations over prolonged periods of time for many years (mean: 15.6 years). The most common occupation was sales representative (17.9%), followed by telephone operator (13.7%), customer service representative (10.5%), health care worker (9.5%), waiter or waitress (5.3%), receptionist (5.3%), and cashier (5.3%). Twenty-nine patients (30.5%) had tardive lingual dystonia. Logistic regression analyses revealed that frequent requirements for professional speaking (*p* = 0.011, odds ratio: 5.66), high stress during work (*p* = 0.043, odds ratio: 5.4), and neuroleptic use (*p* = 0.032, odds ratio: 2.52) were significant contributors to the manifestation of lingual dystonia.

**Conclusion:**

Professions in which conversations in stressful situations are unavoidable may trigger lingual dystonia. Therefore, speech-induced lingual dystonia can be regarded as occupational dystonia in certain cases.

## Introduction

Dystonia is a hyperkinetic movement disorder characterized by sustained or intermittent muscle contractions that cause abnormal, often repetitive, movements, postures, or both (1). Oromandibular dystonia is a focal dystonia involving the masticatory or lingual muscles, and is subdivided into jaw closing dystonia, jaw opening dystonia, jaw deviation dystonia, jaw protrusion dystonia, lingual dystonia, and combinations of these subtypes (2–5). Lingual dystonia is a disabling form of oromandibular dystonia that interferes with important daily activities, such as speaking, chewing, and swallowing; it causes vocational, masticatory, and social disabilities. The involuntary lingual movements vary among repetitive or episodic tongue protrusion or curling and are induced when speaking or eating (6–11). The most common form of involuntary movement is tongue protrusion. Therefore, this condition is referred as lingual (tongue) protrusion dystonia (7, 8, 10). Secondary causes include head injury (12), electrical injury (13), degenerative or inherited diseases (8, 10, 14), and varicella infection (15). Pharmacological therapy for lingual dystonia is impermanent and in most cases only partially effective. The number of studies investigating the effectiveness of botulinum toxin injection has been increasing (6–11, 13, 16–18). Although severe complications, such as significant dysphasia (6), aspiration pneumonia (6), and marked swallowing and breathing difficulties (8), were reported in early studies, in recent years, botulinum toxin therapy has been considered a feasible treatment option for lingual dystonia (10, 11).

Task-specificity is one of the clinical features of focal dystonia. Especially in its early phase, dystonia is often associated with a specific task. Later, the symptoms might extend to other tasks and other body parts and might eventually be present even at rest. Symptoms of oromandibular dystonia often only appear task-specifically, at the time of speaking or chewing. Thus, task-specific dystonia is a form of isolated focal dystonia with the peculiarity of appearing exclusively during performance of a specific skilled motor task. Task-specific focal hand dystonia can affect people who engage in occupations involving repetitive, highly practiced movements. Examples are writer’s cramp, musician’s dystonia, embouchure dystonia, and cramps in golfers, tennis players, and billiards players (19, 20). Additional manifestations arising from occupations have been reported in tailors, shoemakers, and hair stylists (19, 20). This type of dystonia is also called occupational dystonia. Writer’s cramp is the most commonly identified occupational dystonia. Musician’s cramp is characterized by muscle cramps and spasms that occur while playing a musical instrument, such as the piano, guitar, or violin. Embouchure dystonia involves the lips, jaw, and lower cranial muscles of musicians playing brass or woodwind instruments (21).

Except for embouchure dystonia, task-specific occupation-related dystonia in the orofacial region has been reported only in auctioneers (22), bingo callers (23), and those who recite Islamic prayers (24). Previous reports of lingual dystonia have been single case reports (13, 16, 18, 25–33) or case series (6–11). Most reports have been published as Letters to the Editor or Clinical Notes. Therefore, detailed information about these cases is lacking. Patients’ occupations have been reported in the literature only in three cases [bingo caller (23), postal worker (28), and computer engineer (28)]. The aim of the present study was to elucidate phenomenological and clinical characteristics of task-specific lingual dystonia and to assess the relationship between the development of dystonia and occupation as a triggering factor.

## Materials and Methods

### Patients

One hundred forty patients (86 females and 54 males, mean age ± SD: 53.1 ± 17.1 years) with involuntary contracture of the tongue muscle visited our department from 2007 to 2017. Patients who were suspected of having degenerative, inherited, or other neurological diseases were referred to neurologists at our clinic. Patients who had already visited neurologists or neurosurgeons were neurologically examined, and no abnormal findings were revealed. Lingual dystonia was diagnosed based on the characteristic clinical features of focal dystonia, including stereotypy, task-specificity, sensory tricks, morning benefit, and electromyographic findings such as task-specific vigorous bursts during speech (3, 34, 35). Forty-one patients with lingual or orolingual dyskinesia, two patients with psychogenic movement disorder, one patient with neuroacanthocytosis, and one patient with generalized dystonia were excluded from the total of 140 patients. Orolingual dyskinesia refers to repeated, uncontrollable movements such as licking of the lips or chewing-like movements. Patients with psychogenic movement disorder show none of the aforementioned clinical features of focal dystonia; its characteristic feature is inconsistency in the pattern, distribution, and velocity of involuntary movements. Ninety-five patients with task-specific lingual dystonia [53 females (55.8%) and 42 males (44.2%), mean age ± SD: 48.0 ± 14.4 years] were enrolled in this study. Ninety-five age- and gender-matched patients [68 females (71.6%) and 27 males (28.4%), mean age ± SD: 47.2 ± 18.5 years] with temporomandibular disorders were recruited as controls. Temporomandibular disorders involve a number of clinical conditions of the temporomandibular joint, masticatory muscles, and related structures. Temporomandibular disorders were diagnosed according to the Diagnostic Criteria for Temporomandibular Disorders (DC/TMD) (36). The chief complaints of the controls were mild to moderate pain or tenderness in the muscles of mastication.

### Clinical Characteristics and Occupations

The major complaints of the 95 patients with lingual dystonia were dysarthria, masticatory disturbance, dysphasia, discomfort, tongue pain, and esthetic disorder. The clinical features of focal dystonia included stereotypy, task-specificity, sensory tricks, and morning benefit. Sensory tricks are various voluntary maneuvers that ameliorate dystonic postures or movements. Morning benefit refers to the tendency of dystonia to show milder symptoms in the morning. The patients were examined for the clinical features of oromandibular dystonia and associated subtypes, or other dystonia, and asked for histories of neuroleptic drug intake, triggering episodes, consulted departments, and previous diagnoses.

A structured interview was carried out for each patient. The patients were asked for details regarding their occupations, including job description, working time per day and week, load of social or professional interaction, and stress during work. The occupations in which patients had mainly engaged during the 10–20 years before the onset of dystonia were considered for evaluation.

### Analysis

Demographic characteristics, associated movement disorders, subtype of oromandibular dystonia, task-specificity, and sensory tricks were analyzed and compared between idiopathic and tardive dystonia patients. Ratios of occupations were compared between idiopathic and tardive dystonia groups, and between female and male patients.

Multivariate logistic regression models were constructed to estimate the factors that might influence the development of lingual dystonia. Inclusion of variables in a model was according to existing knowledge of risk factors for task-specific dystonia (19). At the beginning, occupational speaking, stress, overwork, neuroleptic use, and gender were selected as five covariates for analysis. Based on the results of structured interviews, the patients and controls were divided into two categories: high requirement for occupational speaking and low requirement for speaking. The high-requirement group included subjects engaged in professions that required them to regularly speak. On the other hand, low-requirement group required no professional speaking. The subjects were also divided into three categories according to stress during work based on the results of the interviews: high stress, normal stress, and no occupational stress. They were divided into three categories according to the amount of work: frequent overwork more than twice a week, normal working time, and no professional work. Psychiatric drugs were taken by 29 patients and 20 controls. Multicollinearity of the variables was checked using the variance inflation factor. The author followed standard methods to estimate sample sizes for multiple logistic regression analyses, with at least 10 outcomes needed for each included independent variable. The covariates were entered into the multivariate regression analysis using maximum likelihood estimation. Odds ratios and 95% confidence intervals were calculated as measures of association.

The chi-square test was used to assess the statistical significance of differences in the distributions. All analyses were performed using SPSS for Windows version 14.0 (SPSS Japan Inc., Tokyo, Japan). The threshold for assigning significance was set at *p* < 0.05.

### Treatments

Patients were treated with a variety of combinations of pharmacotherapy, muscle afferent block therapy (3, 34), botulinum toxin injection (37, 38), splint therapy (39), and a Myomonitor (transcutaneous electro-neural stimulation) device. Subjective improvement was assessed using a linear self-rating scale ranging from 0% (no improvement) to 100% (complete cure).

All patients involved in this study provided written informed consent after receiving a full explanation of the planned treatment. This study was performed in accordance with the Declaration of Helsinki and was approved by the institutional review board and ethics committee of Kyoto Medical Center.

## Results

### Clinical Characteristics

Ninety-five patients with task-specific lingual dystonia (53 females and 42 males, mean age ± SD: 48.0 ± 14.4 years) were analyzed in this study. The demographic characteristics of the patients, associated movement disorders, and subtypes of oromandibular dystonia are summarized in Table 1.

**Table 1:** Patients’ demographic characteristics.

	Total	Idiopathic	Tardive
Number of patients	95	66	29
Age (years) [mean (SD)]	48.0 (14.4)	47.2 (14.6)	49.9 (14.1)
Sex (female, male) [*N* (%)]	53 (55.8%), 42 (44.2%)	34 (51.5%), 32 (48.5%)	18 (62.1%), 11 (37.9%)
Duration of symptom (years) [mean (SD)]	2.7 (3.2)	2.7 (3.4)	2.6 (2.6)
**Associated movement disorders [*N* (%)]**									
Blepharospasm	4 (4.2%)	1 (1.5%)	3 (10.3%)
Cervical dystonia	5 (5.3%)	4 (6.1%)	1 (3.4%)
Writer’s cramp	4 (4.2%)	4 (6.1%)	0
Embouchure dystonia	1 (1.1%)	0	1 (3.4%)
Spasmodic dysphonia	1 (1.1%)	1 (1.5%)	0
Palatal tremor	1 (1.1%)	0	1 (3.4%)
**Subtype of oromandibular dystonia [*N* (%)]**									
Jaw opening dystonia	13 (13.4%)	7 (10.6%)	6 (20.7%)
Jaw closing dystonia	9 (9.5%)	6 (9.1%)	3 (10.3%)
Jaw protrusion dystonia	1 (1.1%)	1 (1.5%)	0
Lip dystonia	1 (1.1%)	1 (1.5%)	0

*No significant differences were observed between idiopathic and tardive patients*.

*N, count*.

Twenty-nine (30.5%) of the 95 patients had been under psychiatric medication and were diagnosed with tardive dystonia. The remaining cases were classified as idiopathic dystonia (69.5%; Table 1). In the 29 tardive dystonia patients, the mean duration of neuroleptic or tranquilizer use was 11.4 ± 7.4 years. Neuroleptic drugs or tranquilizers had been prescribed for depression, schizophrenia, or mood disorders. The prescribed drugs included etizolam (31.0%), aripiprazole (27.6%), flunitrazepam (20.7%), risperidone (17.2%), sulpiride (13.8%), paroxetine (13.8%), olanzapine (10.3%), ethyl loflazepate (10.3%), etc. Twenty of the control subjects also had histories of tranquilizer use or psychiatric medication.

The clinical features are summarized in Table 2. All patients exhibited stereotypical contraction task-specifically during speaking (Table 2). Sensory tricks were exhibited by 60% of the patients, such as chewing gum or candy, touching the jaw with their hands or fingers, a handkerchief, or a mask (Table 2). Other sensory tricks involved a pipe, piece of wood, straw, toothpick, or cotton. Morning benefit was reported by 59 patients (62.1%).

**Table 2:** Comparison of task-specificity and sensory tricks between idiopathic and tardive lingual dystonia.

	Total	Idiopathic	Tardive
**Task-specificity [*N* (%)]**									
Speaking	95 (100%)	66 (100%)	29 (100%)
Chewing	8 (8.4%)	3 (4.5%)	5 (17.2%)
Swallowing	3 (3.2%)	3 (4.5%)	0
**Sensory tricks [N (%)]**	57 (60.0%)	44 (66.7%)	13 (44.8%)
Chewing gum	33 (34.7%)	26 (39.4%)	7 (24.1%)
Candy	7 (7.4%)	4 (6.1%)	3 (10.3%)
Touching with hand	8 (8.4%)	7 (10.6%)	1 (3.4%)
Touching with finger	2 (2.1%)	1 (1.5%)	1 (3.4%)
Handkerchief	2 (2.1%)	1 (1.5%)	1 (3.4%)
Mask	2 (2.1%)	1 (1.5%)	1 (3.4%)
Others	7 (7.4%)	7 (10.6%)	0

*No significant differences were observed between idiopathic and tardive patients*.

*N, count*.

Forty-two patients (44.2%) reported episodes or triggers experienced before onset of symptoms. In 15 patients (15.8%), the triggering episodes involved dental treatment: prosthodontic treatment such as crown, bridge and denture (10.5%), tooth extraction (3.2%), and dental surgery (2.1%). Other triggers included change or cessation of psychiatric medicine (10.5%), excessive stress (9.9%), nasal surgery or examination (3.2%), and tongue training for good pronunciation (2.1%).

All 95 patients had consulted departments or hospitals (mean number ± SD: 3.8 ± 2.3; range: 1–11). The consulted departments included departments of neurology (27.4%), dentistry (20.0%), oral and maxillofacial surgery (17.3%), neurosurgery (11.9%), psychiatry (8.1%), acupuncture (6.5%), otorhinolaryngology (6.5%), internal medicine (1.7%), orthopedics (0.8%), rehabilitation (0.8%), and anesthesiology (0.5%).

Previously, the patients had been diagnosed with or suspected to have conditions of unknown etiology (29.8%), dystonia (14.1%), dyskinesia (11.9%), psychogenic disorders (10.6%), temporomandibular disorders (10.0%), bruxism (4.9%), occlusal problems (2.5%), pericoronitis (1.4%), other conditions (4.1%), or their conditions were considered to be normal (10.7%).

### Occupations

**Figure 1:**
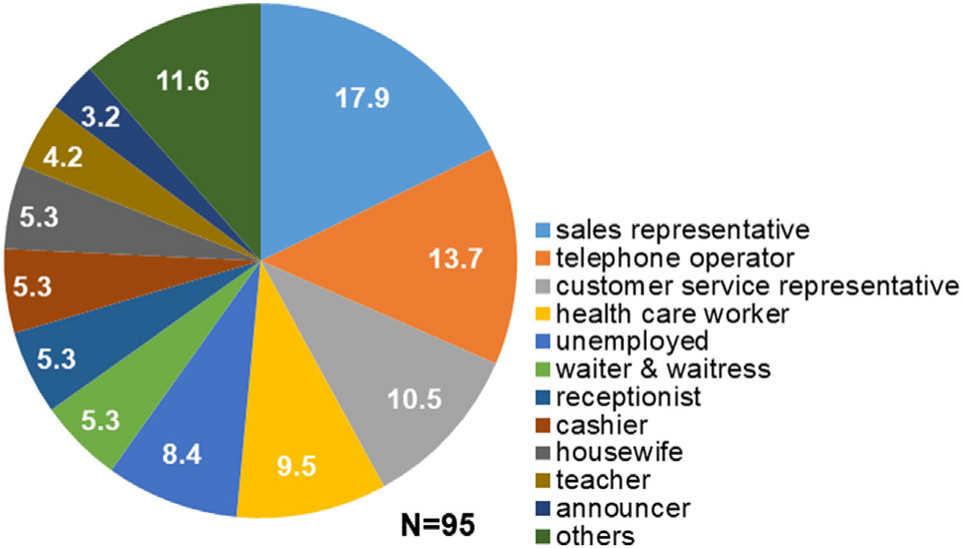
Occupations of patients with task-specific lingual dystonia. The numbers in the pie chart sectors represent percentages. Most of the patients engaged in occupations that required them to talk to customers or other people in stressful situations, such as sales representative, telephone operator, and customer service representative.

Structured interviews revealed that 80 out of 95 patients (84.2%) had regular occupations, and all of them had experienced overtime work. Fifty-nine of the 80 patients (73.8%) reported working overtime more than twice a week. Fifty-one of the 80 patients (63.8%) experienced stress at the workplace. Seventy-eight patients (82.1%) had engaged in occupations that required them to talk to customers or other people over prolonged periods of time. The most frequent occupation among patients with task-specific dystonia was sales representative (17.9%), followed by telephone operator (13.7%), customer service representative (10.5%), health care worker (11.1%: one physician, one pharmacist, one nurse, and six paramedical workers), waiter or waitress (5.3%), receptionist (5.3%), cashier (5.3%), teacher (4.2%), and announcer (3.2%; Figure 1). Others (11.6%) included two train conductors, one site supervisor, one voice actor, one computer engineer, one engineer, one truck driver, one company manager, one part-time jobber, and one architect. Four patients (4.9%) were housewives and 8 (9.9%) were unemployed (Figure 1). The mean duration of occupation was 15.6 ± 11.2 years.

**Figure 2:**
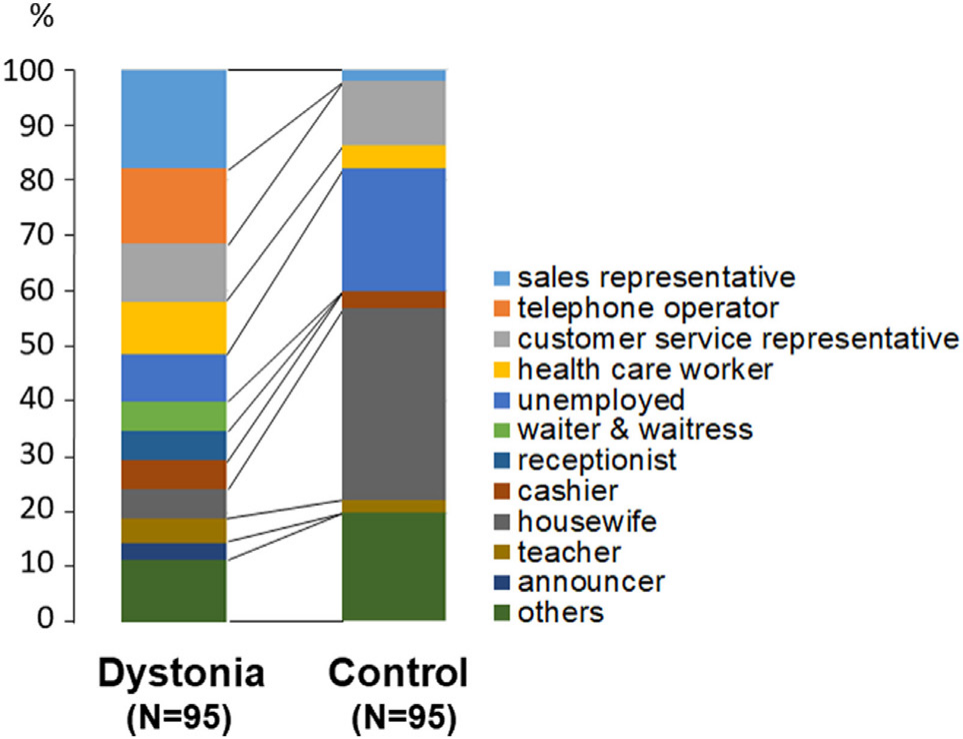
Comparison of occupations between patients with lingual dystonia and controls. Housewives (*p* < 0.01) and unemployed persons (*p* < 0.05) were significantly more prevalent among the controls than among the dystonia patients.

Differences in the distribution of occupations between patients and controls are shown in Figure 2. There were significantly more housewives (*p* < 0.01, chi-square test) and unemployed persons (*p* < 0.05) among the controls than among the dystonia patients.

Occupations in the high-requirement group (*N* = 78 patients and *N* = 23 controls) were sales representative, telephone operator, customer service representative, health care worker, and so on. On the other hand, most of subjects in low-requirement group (17 patients and 72 controls) were unemployed or housewives. The subjects were also divided into three categories according to stress during work: high stress group (51 patients and 9 controls), normal stress group (31 patients and 25 controls) and no occupational stress group (13 patients and 61 controls). They were divided into three categories according to the amount of work: frequent overwork group (59 patients and 20 controls), normal working time group (23 patients and 4 controls), and no professional work group (13 patients and 61 controls). Psychiatric drugs were taken by 29 patients and 20 controls, and unused by 66 patients and 75 controls.

The results for significant covariates in the multivariate regression analysis are summarized in Table 3. Logistic regression analyses indicated that frequent requirements for professional speaking (*p* = 0.011, odds ratio: 5.66), high stress during work (*p* = 0.043, odds ratio: 5.4), and neuroleptic use (*p* = 0.032, odds ratio: 2.52) were significant contributors to the manifestation of lingual dystonia.

**Table 3:** Results of a multivariate logistic regression analysis, in which the outcome is presence or absence of lingual dystonia.

Independent variable	Regression coefficient	Standard error	*p*-Value	Odds ratio	95% confidence interval
High speech requirement	1.734	0.685	0.011	5.661	1.477–21.697
High stress during work	1.686	0.835	0.043	5.4	1.051–27.747
Neuroleptic use	0.925	0.430	0.032	2.522	1.085–5.865

Among the group of patients with idiopathic dystonia, sales representative and telephone operator tended to be more frequent than in the tardive dystonia group. However, the difference did not reach statistical significance. Among female patients, telephone operators and receptionists were more common than among male patients, whereas sales representatives were more frequent among male patients than among female patients. However, these differences were not significant. There were no significant differences in demographic or clinical features between idiopathic and tardive dystonia patients (Tables 1 and 2).

### Treatments

The author prescribed trihexyphenidyl, baclofen, clonazepam, tiapride, zolpidem, or Chinese medicine to 52 patients (54.7%). The mean subjective improvement from pharmacotherapy was 25.9%. Muscle afferent block therapy (3, 34) was performed in 53 patients (55.8%) for a total of 369 times (range 1–31 times), with mean subjective improvement 30.3%. Botulinum toxin was injected into the genioglossal muscle in 62 patients (65.3%), 306 times (range 1–15 times), without significant complication (36, 37). Other injected muscles included the lateral pterygoid, masseter, temporalis, medial pterygoid, and digastric muscles, depending on the symptoms of each patient. The mean subjective improvement from botulinum toxin therapy was 72.3%. Occlusal splints (39) were fabricated and inserted into the mandibular dental arch in 28 patients (29.5%). The splints were inserted during daytime. The mean subjective improvement rate for splint therapy was only 22.5%. The Myomonitor device was applied for several patients with jaw elevator muscle pain. The full data obtained from the various treatment methods cannot be reported in this paper because of space limits. Therefore, detailed results and a discussion of these findings for each treatment method will be published elsewhere.

## Discussion

To the best of the author’s knowledge, the present study is the first to report that occupations which require workers to talk in stressful situations are plausible triggering factors for task-specific lingual dystonia in predisposed individuals. Speech-induced lingual dystonia may, therefore, be a type of occupational dystonia.

### Pathophysiology of Task-Specific Dystonia

Isolated focal task-specific dystonia impairs not only motor dexterity, but also somatosensory perception in extremely well-trained and skilled motor tasks. There are two dominant hypotheses regarding the underlying mechanisms of isolated focal task-specific dystonia: impaired inhibition and abnormal plasticity regulation. Early studies suggested abnormal inhibitory processes in the motor and somatosensory systems of patients with task-specific dystonia (40, 41). Accordingly, inhibition has been shown to be reduced in multiple intracortical (42) and cortico-cortical networks (43). The second hypothesis, posing that task-specific dystonia results from plasticity dysregulation in the brain (44), is supported by data showing that plasticity in patients with dystonia is excessive in magnitude and topographically unspecific (43). A complex interaction between over-practice, genetic predisposition, and other biomechanical and psychological factors appears to underlie the loss of inhibition and the plasticity-driven reorganization of somatotopic representations within sensorimotor regions of patients with focal task-specific dystonia (45). The abovementioned studies are based on results from patients with focal dystonias such as writer’s cramp. We previously attempted to elucidate the cortical neurophysiology related to jaw movements and perception in the stomatognathic system (46–51), including the tongue (49, 50) and the palate (48), using electroencephalography (46, 47) and magnetoencephalography (48–50). Recordings using neuroimaging and non-invasive brain stimulation techniques are difficult in patients with oromandibular dystonia, owing to artifacts arising from masticatory muscle activity and involuntary jaw movements. Several studies of cortical activity in oromandibular dystonia have been published (51), but further studies are required to elucidate the pathophysiology, neurophysiology, and etiology of task-specific lingual dystonia.

### Prevalence of Lingual Dystonia

The overall prevalence of primary dystonia was calculated in a meta-analysis as 164.3 per million (52). Prevalence estimates of task-specific dystonia in the general population range from 7 to 69 per million (53, 54). The prevalence of oromandibular dystonia was estimated at 68.9 per million (55). To date, prevalence estimates for lingual dystonia have not been reported. Lingual dystonia has previously been described mostly in single case reports (13, 16, 18, 25–33) or case series (6–11). Thus, methodologically sound studies of lingual dystonia are lacking. To obtain significant evidence, further studies with much larger sample sizes are required. To the author’s knowledge, this is the largest series of lingual dystonia cases reported so far. Lingual dystonia is considerably less common than other types of focal dystonia; in particular, isolated task-specific lingual dystonia is extremely rare, even at movement disorder clinics or the neurological departments of university hospitals. Only 14.1% of all patients in this study had been diagnosed with dystonia before visiting our department. Many patients had been previously misdiagnosed as having dyskinesia (11.9%), psychogenic disorders (10.6%), or temporomandibular disorders (10.0%); or remained undiscovered as having an unknown etiology (29.8%) or being normal (10.7%). Most patients, after being misdiagnosed, probably abandon further consultation. Thus, the actual prevalence of isolated task-specific lingual dystonia is likely much higher than currently thought.

The population in the present report differs significantly from previous studies, which mostly reported patients with generalized dystonia, secondary to degenerative, inherited, or post-encephalitic diseases, or neuroacanthocytosis (8, 10, 14). By contrast, in this study, all 95 patients had isolated, task-specific lingual dystonia. Secondary and generalized cases were excluded from the analysis. The author’s department does not specialize in neurology, but rather oral and maxillofacial surgery. Patients with hyperkinetic involuntary movements of the tongue were referred to the author, particularly patients with isolated lingual dystonia who came to visit our department from far away. The author has established a web site for patients with oromandibular dystonia, which has been accessed by more than one million visitors from over 190 countries and regions all over the world (37). At the author’s department, we offer a comprehensive range of multimodal therapies for involuntary movements of the orofacial region, including medication, muscle afferent block therapy (3, 34), botulinum toxin therapy (37, 38), splint therapy (39), Myomonitor, and surgery (35, 56). Many patients with involuntary movements are referred to the author from all over Japan and, indeed, the world. The numerous patients who had already abandoned consultation might have visited the author’s clinic after informing themselves about oromandibular dystonia *via* the author’s web site.

### Occupation As Triggering Factor for Lingual Dystonia

In this study, structured interviews revealed that most of the patients with task-specific lingual dystonia were engaged in professions that required frequently speaking to other people over long periods of time in highly stressful situations. Sales representative was the most frequent occupation among the patients. Customer service representatives, telephone operators, waiters or waitresses, receptionists, and cashiers also have to engage in conversations with customers over long periods of time. Similarly, health care workers (physician, nurse, and paramedical staff) and teachers (professor or schoolteacher) must talk to, explain to, and teach patients or students over prolonged periods of time, often under stressful conditions. Many of the patients participating in this study complained about overtime work in tense and stressful environments. They were often required to work overtime to reach higher levels of performance. In these patients, increased working hours, psychological stress, and task difficulty might have contributed to the development of lingual dystonia. In this study, a high requirement for occupational speaking (odds ratio: 5.66) and high stress during work (odds ratio: 5.4) were significantly related to lingual dystonia. Overwork, however, was not significant. Stress accompanying an occupation might be more relevant to the development of lingual dystonia than overwork. Further studies more precisely evaluating stress and overwork from a psychiatric standpoint are necessary. In order to be associated with dystonia, a task needs to have been performed over a sufficient amount of time, as evidenced by a study of the occurrence of dystonia in occupational tasks (19). In that study, the mean duration of employment in the occupation was 15.6 years.

Adult-onset focal primary dystonia is generally most common in females (53, 54), while task-specific dystonia may be more prevalent in males. In the current study, a trend toward female predominance (55.8%) was observed among the study population. We did not observe a significant difference in clinical characteristics and occupations either between idiopathic and tardive patients, or between female and male patients. A previous study reported no differences in the demographic characteristics between tardive and idiopathic groups in patients with oromandibular dystonia (57). However, in this study neuroleptic use was a significant factor (odds ratio: 2.52) related to lingual dystonia. Psychiatric drugs might influence the development of lingual dystonia as a type of triggering factor other than stress or high requirement for speech. Population-based normal controls, and data on occupations in other dystonias such as cervical dystonia, are required to confirm this hypothesis. As the selection of subjects and controls was clinic-based in this study, an influence of referral bias cannot be excluded. A study concerning occupations in Parkinson’s disease patients suggested that occupational studies may be susceptible to referral bias because social networks may spread preferentially *via* jobs (58). Further studies involving larger population-based samples are needed to fully elucidate this relationship.

### Other Triggering Factors

Risk factors for task-specific dystonia include environmental factors such as specific task requirements and parameters of task reproduction, non-task-related factors, such as injury and personality, psychological factors, and genetic factors (19).

The influence of task difficulty, and the requirement for precision or a need to perform while avoiding errors, is similarly risk factors (19). Anxiety and personality types such as extreme perfectionism are elevated in musicians’ dystonia (59, 60). Announcers and voice actors need precise wording, intonation, and pronunciation to ensure that their performance meets high expectations. The pathophysiologic factors or mechanisms responsible in such cases might be the same as those underlying musicians’ dystonia.

Trauma is also thought to be a risk factor for task-specific dystonia. Facial injury can precipitate dystonia, manifesting as embouchure dystonia (20). It has been suggested that orofacial trauma, including dental procedures, may precipitate the onset of oromandibular dystonia in predisposed individuals (61). In this study, 18.5% of patients had received dental treatment. Perhaps certain risk factors need to accumulate over time, or in combination, to initiate dystonia (19).

In addition to occupation, other pathophysiological factors or mechanisms, including environmental influences and genetic factors, need to be considered. Approximately 10–20% of cases of task-specific dystonia have a positive family history (62). The preponderance of males developing task-specific dystonia, and a positive family history of musician’s dystonia, suggests an influence of genetic risk factors (63, 64). A familial case has been reported for speech-induced tongue protrusion dystonia (65). In future studies, the family histories of patients should be comprehensively investigated. In addition, genetic tests may be needed to elucidate the influence of genetic factors on task-specific dystonia.

## Conclusion

Most patients with task-specific lingual dystonia engaged in professions that required them to regularly speak in stressful situations for long periods of time (telephone operator, sales, and customer service representative). The author suggests that speech-induced lingual dystonia can be considered a kind of occupational dystonia.

## Ethics Statement

All patients involved in this study provided written informed consent after receiving a full explanation of the planned treatment. This study was performed in accordance with the Declaration of Helsinki and was approved by the institutional review board and ethics committee of Kyoto Medical Center.

## Author Contributions

The author diagnosed and treated all patients, analyzed the results, and wrote the manuscript.

## Conflict of Interest Statement

The author declares that the research was conducted in the absence of any commercial or financial relationships that could be construed as a potential conflict of interest.
